# Surveillance of *Wuchereria bancrofti* infection by anti-filarial IgG4 in urine among schoolchildren and molecular xenomonitoring in Sri Lanka: a post mass drug administration study

**DOI:** 10.1186/s41182-019-0166-5

**Published:** 2019-06-13

**Authors:** Hidekazu Takagi, Thishan C. Yahathugoda, Bumpei Tojo, Upeksha L. Rathnapala, Fumiaki Nagaoka, Mirani V. Weerasooriya, Makoto Itoh

**Affiliations:** 1 Department of Microbiology & Immunology, Aichi Medical University School of Medicine, Nagakute, Aichi 480-1195 Japan; 2 School of Tropical Medicine and Global Health, Nagasaki University, 1-12-4 Sakamoto, Nagasaki, 852-8523 Japan; 3 Filariasis Research Training and Service Unit (FRTSU), Department of Parasitology, Faculty of Medicine, University of Ruhuna, Galle, Sri Lanka

**Keywords:** *Wuchereria bancrofti*, *Culex* (*Cx*) *quinquefasciatus*, PCR, Urine, Anti-filarial IgG4, Schoolchildren, Molecular xenomonitoring (MX)

## Abstract

**Background:**

Surveillance of hidden foci or resurgence of the bancroftian filariasis has high priority to maintain the elimination status in Sri Lanka. For the surveillance, two methods were applied in Matotagama, Matara, Sri Lanka; (i) molecular xenomonitoring (MX) by PCR to detect parasite DNA in the vector, *Culex* (*Cx*) *quinquefasciatus* and (ii) survey of anti-filarial IgG4 in urine samples from schoolchildren.

**Results:**

Mosquitoes were collected monthly from index houses for 17 months (2013 to 2014) to confirm the existence of bancroftian parasite. Index houses in Matotagama had recorded microfilaria-positive cases in the recent past. Five schools were selected considering Matotagama as the catchment area and all students who presented on the day were tested for urine anti-filarial IgG4 in 2015. *Wuchereria bancrofti* DNA in *Cx*. *quinquefasciatus* pools were found in 14 of 17 months studied and ranged between 0 and 1.4%. The MX rate was greatly increased at least two times in the year following the driest months (March, August). A total of 735 schoolchildren were tested for urine anti-filarial IgG4. Three schools located closer to the MX area had higher positive rates, 3.4%, 3.6%, and 6.6%. Both highest positive rates of MX and urine were located in a nearer vicinity.

**Conclusion:**

Monthly collections to study lymphatic filariasis (LF) transmission by MX was conducted for the first time in Sri Lanka. We observed that the filarial DNA-positive rate had an association with seasonal cycle of precipitation. More than 1% filarial DNA and > 5% anti-filarial antibody rates confirmed ongoing transmission in Matotagama. The combination of two non-invasive surveys, the urine anti-filarial IgG4 levels of schoolchildren and MX of vector mosquitoes, would be a convenient package to monitor the ongoing transmission (hotspots) of LF in the surveillance.

## Introduction

Lymphatic filariasis (LF) impairs the lymphatic system and runs a chronic course of illnesses. Eight hundred and fifty-six million people in 52 countries worldwide remain threatened by LF and require preventive chemotherapy (PCT) to stop the spread of this parasitic infection [1]. World Health Assembly resolution WHA50.29 encouraged Member States to eliminate LF as a public health problem. In response, Global Programme to Eliminate LF (GPELF) was launched by World Health Organization (WHO) in 2000 [2]. In 2002, national Programme to Eliminate LF (PELF) of Sri Lanka was initiated and laid the target to eliminate LF by 2020 [1]. In PCT, the main strategy implemented was a combined dose of diethylcarbamazine citrate (DEC) (adult—300 mg; 2–12 years—150 mg) and albendazole (400 mg) given annually to every individual with 100% geographical coverage and over 80% drug coverage [3]. In Sri Lanka, eight endemic districts of southern, western, and north-western provinces received five to eight rounds of this treatment, mass drug administration (MDA) to stop transmission. In 2016, WHO has declared that Sri Lanka had eliminated LF as a public health problem [4].

Although many countries including Sri Lanka have achieved remarkable reductions in microfilaria (mf)-positive patients after careful PCT, there were cases reported even after WHO certification of LF elimination [5]. WHO has foreseen the issues that a national campaign could have at near elimination or post-elimination period. In response to these problems, WHO announced its policy and strategy for the next 10 years in 2011 [6]. Previously, national campaigns were asked to use immunochromatography test (ICT) for filarial antigen detection kit in schools or community-based samples. However, drawbacks associated with ICT card-test [7] have been removed by the recent introduction of more sensitive filariasis test strip [3]. As such antigen-based tests remained more reliable test for detecting ongoing transmission. However, the GPELF realized that the assessment of ongoing transmission or resurgence of infection could not be achieved by antigen testing alone. Therefore, a PCR-based molecular xenomonitoring (MX) was introduced [8]. Mosquito infection rate is a good indicator to monitor ongoing LF transmission or resurgence. MX in *Culex* (*Cx*) *quinquefasciatus* transmitted LF can be carried out with Gravid Trap-based mosquito collections where the disturbance to residents is minimum. MX on Gravid Trap caught mosquitoes would be an effective method to confirm LF elimination in Sri Lanka where only *Cx*. *quinquefasciatus* transmitted LF is present. On the other hand, several investigations have emphasized the usefulness of antibody detection technique for prevalence, post MDA surveillance, and verification studies [9–16]. Itoh et al. have validated a similar antibody-based test as a diagnostic test using sample of urine [17]. Since then, urine enzyme-linked immunosorbent assay (ELISA) has been tested in several countries including Sri Lanka and further validated the test sensitivity and usefulness [3, 12, 18–21]. As such, we believe that urine ELISA is a more suitable tool to investigate true elimination or resurgence especially in the Sri Lankan context. Urine ELISA is acceptable due its easy sample collection with much improved compliance, especially among schoolchildren [12, 22]. With these features, the combination of “urine and mosquito” based tools are useful in the assessment of ongoing LF transmission in hotspots, especially at the last stages of a national elimination programme.

In this study, we applied urine ELISA to examine the anti-filarial IgG4 in urine samples from schoolchildren, and PCR to detect the filarial DNA in vector mosquito, *Cx*. *quinquefasciatus* in Matotagama, Matara as a surveillance package.

## Results

### Anti-filarial IgG4 among schoolchildren

**Fig. 1:**
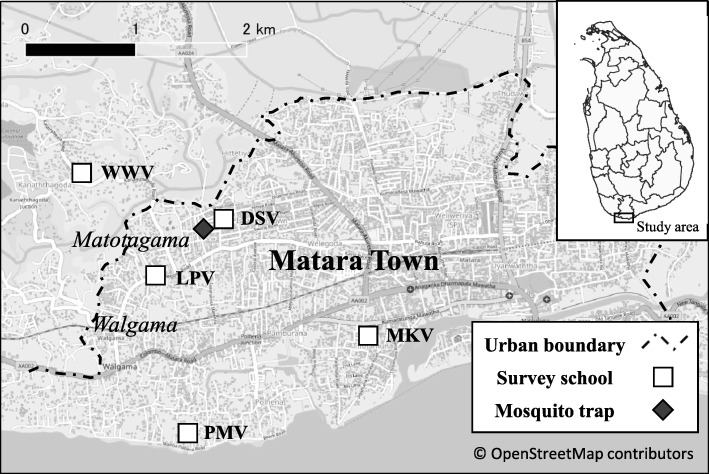
Location of surveyed schools, mosquito trap,s and spatial distribution of anti-filarial IgG4 values in Matara. The map shows the location for survey area: Matara is located in the southern part of Sri Lanka. Five schools (DSV, LPV, WWW, PMV, and MKV) surveyed for schoolchildren IgG4 are shown as white box. Black diamond indicates the location of CDC Gravid Traps for collecting *Cx*. *quinquefasciatus*. The center coordinates of the eight Traps are approximately N5.954128, E80.522119

**Fig. 2:**
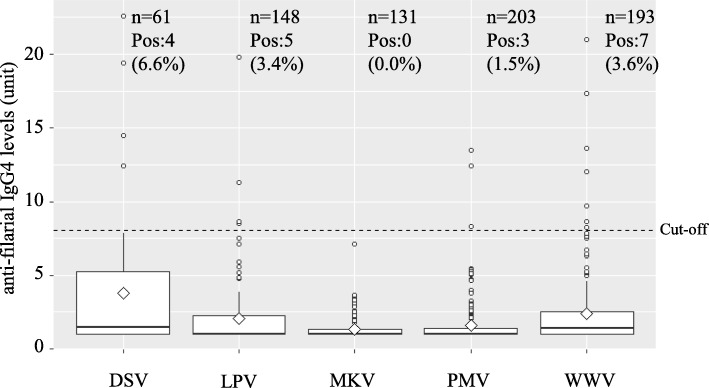
Anti-filarial IgG4 levels of schoolchildren from five schools surveyed. Date shows that the anti-filarial IgG4 antibody levels in urine of schoolchildren belonging to each school (DSV, LPV, MKV, PMV, WWW). Total number of schoolchildren (*n*), number of anti-filarial IgG4 positives (Pos), and mean (white diamond), median (horizontal bar) were indicated. The dash line indicates the cut off value (7.08 U)

A total of 735 urine samples from five schools were examined for anti-filarial IgG4 (Fig. 1). Arbitrarily abbreviations (LPV; PMV; WWV; MKV; DSV) were given to selected schools to obscure the identity of the school. The results are summarized in Fig. 2. The highest prevalence was recorded in DSV (6.5%). Pairwise comparisons using Wilcoxon rank sum test (*p* value adjustment method: Bonferroni) was performed (Table 1) as these five distributions were not normally distributed (*p* = 2.2 e-16, by Shapiro-Wilk normality test) and a significant difference in medians (*p* = 3.2 e-11, Kruskal-Willis rank sum test). According to the test results shown in Table 1, two categories of schools can be identified: (i) low IgG4 level group (MKV; 0% and PMV; 1.5%) and (ii) high IgG4 level group (LPV; 3.4% and WWV; 3.6%, and DSV; 6.6%) (*P* < 0.05).

**Table 1:** Pairwise comparisons using Wilcoxon rank sum test

	DSV	LPV	MKV	PMV
LPV	0.0827			
MKV	1.9e-05	0.0051		
PMV	1.2e-05	0.0072	1.0000	
WWV	0.9430	1.0000	2.1e-06	8.7e-07

*P* value adjustment method: Bonferroni

### Molecular xenomonitoring

In all, a total of 788 pools (14,338 mosquitoes) of blood-fed or gravid mosquitoes were tested for filarial DNA by PCR. The results were summarized in Table 2. *Wuchereria bancrofti* DNA was detected in mosquito pools collected in 14 out of 17 months. More than 0.25% of the actual DNA-positive rate was observed during 8 out of 17 months. Actual DNA-positive rates ranged between 0.00 and 1.43% while recording higher values in month of April (0.93%) and September (1.43%), 2013. When CI upper limit was considered, the actual DNA-positive rates exceeded 1% of endpoint target in 7 out of 17 months.

**Table 2:** Filarial DNA rate of mosquitoes collected in Matotagama from February 2013 to June 2014

Date	Captured mosquitoes	Num. of pools^a^	Num. of positive pools	% of positive pools	Filarial DNA positive rates in mosquitoes^b^		
					Estimation (%)	Lower (CI)	Upper (CI)
2013/2/25	904	49	2	4.082	0.224	0.040	0.733
2013/3/26	495	28	1	3.571	0.202	0.012	0.983
2013/4/27	806	43	7	16.279	0.931	0.414	1.838
2013/5/18	710	40	1	2.500	0.141	0.008	0.684
2013/6/26	958	49	0	0.000	0.000	0.000	0.385
2013/7/27	1346	71	1	1.408	0.074	0.004	0.360
2013/8/31	601	34	2	5.882	0.339	0.061	1.112
2013/9/19	865	47	11	23.404	1.433	0.762	2.494
2013/10/26	838	45	3	6.667	0.367	0.097	0.994
2013/11/30	796	43	3	6.977	0.386	0.102	1.048
2014/1/4	403	25	2	8.000	0.510	0.092	1.680
2014/2/2	936	48	3	6.250	0.327	0.087	0.884
2014/2/21	1534	82	1	1.220	0.065	0.004	0.316
2014/3/21	917	55	0	0.000	0.000	0.000	0.403
2014/4/25	960	59	1	1.695	0.104	0.006	0.506
2014/5/30	1009	54	5	9.259	0.513	0.192	1.133
2014/6/7	260	16	0	0.000	0.000	0.000	1.300

^a^*Cx*. *quinquefasciatus* mosquitoes were captured by gravid traps at eight indexed houses and pooled in tubes (1–20 mosquitoes/pool)

^b^Point estimates and CIs of filarial DNA rate of mosquitoes were calculated using this binGroup library (pooledBin function) of R software

### Statistical analysis of correlation among precipitation, number of captured mosquitoes, and filarial DNA-positive rates of mosquitoes

**Fig. 3:**
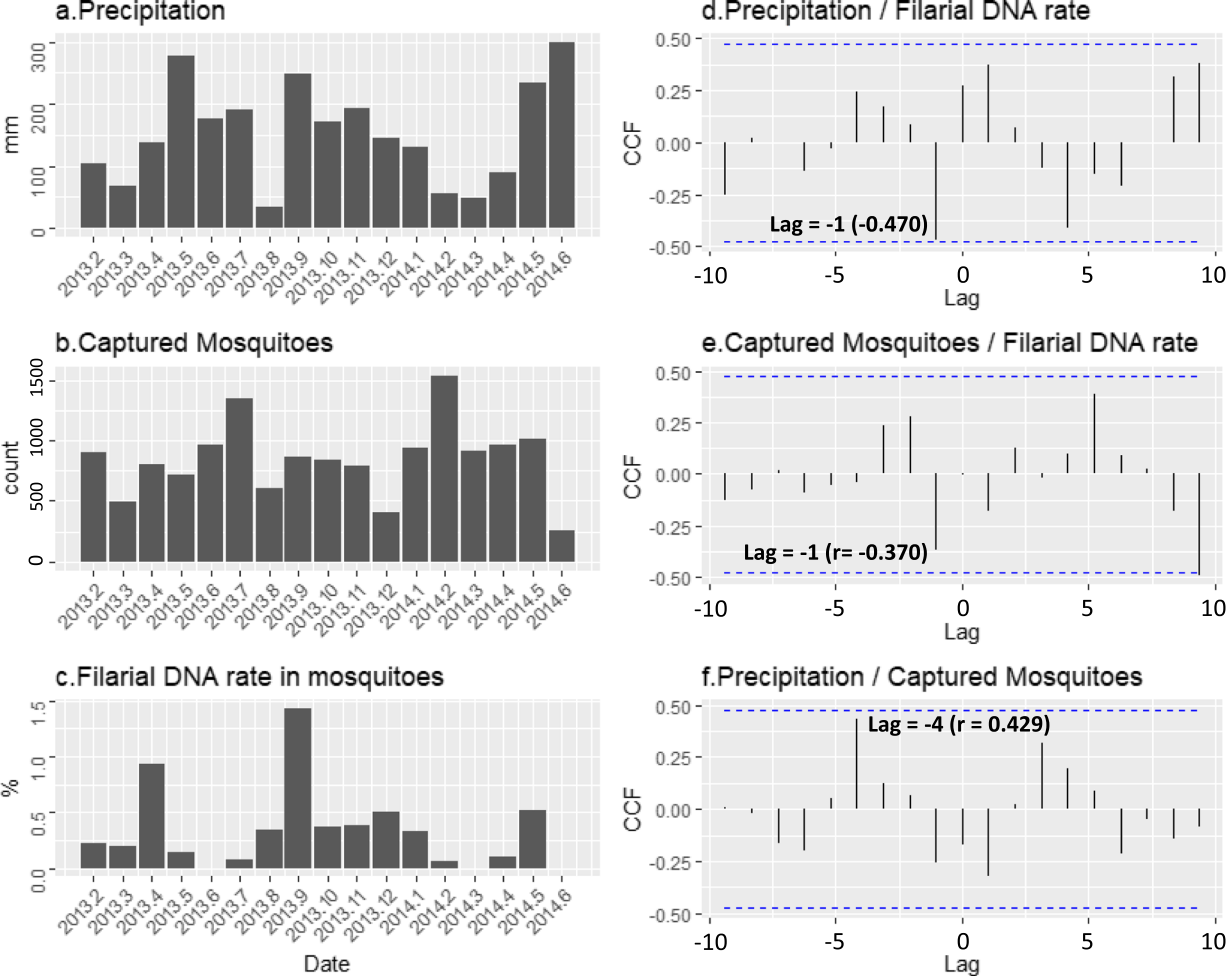
Seasonal fluctuation of precipitation, number of captured mosquitoes, filarial DNA rate in mosquitoes and results of their statistical analysis. The left column of figure illustrates Data of precipitation (**a**), number of captured mosquitoes (**b**), and filarial DNA rate in mosquitoes (**c**) between February 2013 and June 2014. The right column shows partial autocorrelation of precipitation data using R autoplot function (**d**), cross-correlation coefficient (CCF) between precipitation and filarial DNA rate (**e**), and between captured mosquitoes and filarial DNA rate (**f**) using R autoplot function

Monthly fluctuations among precipitation, number of captured mosquitoes, and their filarial DNA-positive rates during the study period are summarized in Fig. 3. Factors affecting the change of filarial DNA-positive rate were analyzed by calculating partial autocorrelation coefficient (partial ACF) of these parameters itself and cross-correlation (CCF) between these parameters (Fig. 3d–f). Focusing on the number of lags with the highest ACF of each variable using R autoplot function, a partial autocorrelation test (Box-Ljung Test) was performed to judge the time dependency. From the test results, among the highest partial ACF of these parameters, the precipitation had a partial autocorrelation with lag = 3 (*r* = − 0.458 and *p* = 0.0904), but the mosquito-captured population (lag = 4) and the mosquito DNA-positive rate (lag = 6) did not have partial autocorrelation (*r* = − 0.405 and − 0.345, *p* = 0.4279 and 0.6807). Furthermore, CCF was analyzed for each combination of precipitation, filarial DNA-positive rate, and captured mosquito population using R autoplot function. Precipitation and mosquito population were logarithmically converted for this analysis. Considering the life span of mosquitoes, it is difficult to think about the actual correlation to the high CCF in long lags. Therefore, only the lag of about 2–4 months of each CCF was paid attention in this paper. A negative CCF (*r* = − 0.470) was observed with lag = − 1 between precipitation (1 month ago) and filarial DNA-positive rate (Fig. 3d). A negative CCF (*r* = − 0.370) with lag = − 1 between captured mosquito population (1 month ago) and filarial DNA-positive rate, and positive CCF (*r* = 0.429) with lag = − 4 between precipitation (4 months ago) and mosquito population, also be observed (Fig. 3e, f). The Pearson product-moment correlation (Pearson’s *r*) was calculated using R cor.test function on these three combinations of variables. However, since these variables are unit-root processes (*p* value = 0.257, 0.261, 0.067 by Phillips-Perron test using R PP.test function), Pearson’s *r* were calculated after applying the difference operator to the series to avoid “spurious regression.” From the Pearson’s correlation result, there was statistically significant correlation (*r* = − 0.767, *p* = 0.0008) between the precipitation decreasing of previous month and the filarial DNA-positive rate increasing. Mosquito density could be seasonal factored into precipitation, but could not find clear statistical correlation (*r* = 0.507, *p* = 0.09).

## Discussion

### Effectiveness of the surveillance package consist of urine ELISA and MX strategies

From 2002 to 2006, the national PELF in Sri Lanka had conducted annual MDA and many reports of its evaluation were published [22–25]. Sri Lanka had easily passed transmission assessment surveys (TAS) in selected sentinel and spot check sites, as such WHO has formally acknowledged the elimination of lymphatic filariasis in Sri Lanka as a public health problem in 2016 [4]. However, there were a few areas apparently smaller than original size of evaluation units (EUs) with mf rate of more than 1% for *W*. *bancrofti*, which is higher than the endpoint target [5]. We have also shown residual mf in Hamugewatta and Matotagama[sd] during post-MDA surveys conducted in 2008, 2009, 2012, and 2014 [26]. Further, we observed that the positive cases were accumulated in a smaller geographical area. Therefore, in the current survey, the EU size was reduced by a different epidemiological approach and we located a smaller focus of ongoing transmission. Mf-positive signals were considered in the sample selection for MX. We used eight CDC gravid traps in eight selected houses with recent history of mf-positive signals. Six months out of 17 months surveyed have recorded over 1% (upper limit of 95% CI was considered) of WHO recommended MX endpoint target (Table 2) [25]. Due to inherent limitations of MX, having only MX results for the confirmation of ongoing transmission in an area is not accurate. Capacity of the vector to transmit the L3 parasite load cannot be directly measured by MX. The longevity of the vector could be an issue. Therefore, we combined MX data with anti-filarial IgG4 to justify the true ongoing transmission. Anti-filarial antibody prevalence in first and second grade primary schoolchildren could be a good maker for recent exposure and endpoint target would be 5% [5]. Hamugewatta and Matotgama[sd] were the catchment area for schools selected for the urine-based anti-filarial antibody testing. Urine-based IgG4 against recombinant SXP1 has provided highly sensitive and specific results in Sri Lanka [3, 17]. Three schools (DSV, LPV, and WWM) closer to the MX study area have recorded higher urine anti-filarial antibody-positive rates than other two schools situated further away from the said area (Figs. 1 and 2). The closest school (DSV) recorded more than 5% anti-filarial antibody-positive rates which is significantly higher compared to that of schools further away (Table 1). As such, we can confirm the ongoing transmission in this smaller EU by using both human and vector parameters.

### The importance of having a seasonal schedule for MX surveys in Sri Lanka

This was the first occasion that seasonal variation of DNA of *W*. *bancrofti* in vectors was examined in Sri Lanka. A similar seasonal variation in *Cx*. *quinquefasciatus* biting and bancroftian filariasis transmission was described in elsewhere [27]. In order to interrupt filariasis transmission, vector infection must be suppressed below an endpoint target to ensure that no new infection occurred. WHO recommended to collect around 10,000 mosquitoes from 250 household from an implementation unit to show the estimate is below the endpoint target (0.25%) [28]. However, the endpoint targets of MX in post-MDA surveillance for *Cx*. *quinquefasciatus* are likely to be different in different countries because of the heterogeneity of the vector–parasite relationship [5]. Therefore, when we study a smaller locality (portion of an implementation unit), the proposed sampling method could be utilized. We were able to detect target prevalence with more than endpoint during 8 out of 17 months by using index house sampling (Table 2). Therefore, a similar approach could be used to carry out in minor foci to assess the ongoing transmission.

It is noteworthy that the MX picks all development stages of *W*. *bancrofti* which include microfilariae, L1, L2, and infective larval stage (L3). Hence, the direct calculation of infectivity rate is impossible as we obtained from the mosquito *Cx*. *quinquefasciatus* dissections [29, 30]. Therefore, we used a mathematical model developed by Christopher and others (details are described in methods) to approximate the actual infection rate using MX-positive pool numbers.

The local environmental conditions also affect the transmission: rainfall, temperature, humidity, and soil type can also affect the production of breeding sites and the survival of adult mosquitoes [31–34]. Southern province of Sri Lanka has the seasonal cycle of rainy seasons: First inter-monsoon (March–April), Southwest-monsoon (May–September), Second inter-monsoon (October–November), and Northeast-monsoon (December–February). The filarial DNA-positive rate of mosquitoes in the area covered greatly increased at least two times in the year (Fig. 3c, e). This occurred in the month following the driest month (March, August). The span of the current survey is insufficient to come to a conclusion; more studies should look into seasonality of bancroftian filariasis transmission in Sri Lanka to assess the reproducibility of the data presented. Further, confounding factors other than seasonality should be studied to understand the greatly increased bancroftian DNA-positive rate in *Cx*. *quinquefasciatus* in some months of the year. Increase in filarial DNA-positive rate may be explained by numbers taking blood meals is more with increase in vector populations. Correlation between the precipitation of the previous month and the higher frequency of mosquito blood sucking behavior generate a new research area and need in-depth research. Present results suggest that the seasonal schedule of MX for lymphatic filariasis in Sri Lanka should be determined by considering at least seasonal cycles of precipitation to avoid the underestimation of the filarial DNA-positive rate. By the Pearson’s test, a significant correlation was not observed between the mosquito population and filarial DNA-positive ratio (*r* = 0.308, *p* = 0.26). No significant ACF was observed in the population of mosquitoes. Therefore, it is difficult to predict the time when the mosquito populations would decrease and it is difficult to consider mosquito population changes in the MX survey scheduling. This may be due to the inadequacy of the cyclic data in the present study.

The national anti-filariasis campaign of Sri Lanka collected mosquitoes for MX by keeping gravid traps 1–3 consecutive nights in selective zones of an EU [5]. Since our study has documented possible seasonal variation in DNA-positive rate in mosquitoes, we would like to recommend sample collections with the start of the Southwest-monsoon (April and May) and at end of the Southwest-monsoon (September and October). It is noteworthy that we observed 3 months (March, June 2013 and 2014) with zero MX results from a hotspot (Fig. 3). Results suggest dry months (e.g., March) and months with heavy storms (e.g., June) should be skipped for conducting MX surveys.

Most of the sessional studies related to vector-borne diseases were conducted more than 36 months [35, 36]. The present study has collected samples for MX only for 17 months. Therefore, a true analysis of sessional variation is not possible. Further, the optimal number of collections per year to yield best results has not been tested in the present study. As such, additional studies by including much wider sessional seasonal studies and to confirm the reliability of the data presented in this study will be needed.

**Fig. 4:**
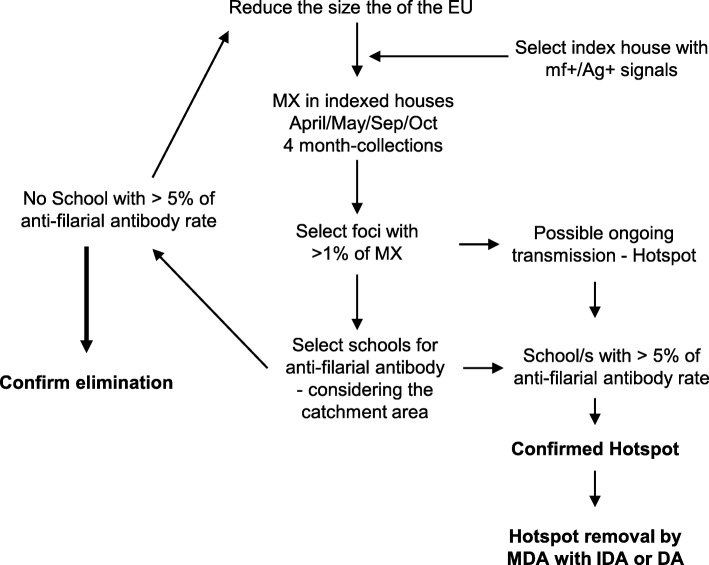
A convenient epidemiological approach to find hotspots of LF transmission and action plan for hotspot removal in Sri Lanka. *EU* evaluation units; *mf*+/*Ag*+ microfilaria positive and/or filarial antigen positive; *MX* molecular xenomonitoring; *MDA* mass drug administration; *IDA* a combination of ivermectin, diethylcarbamazine, and albendazole; *DA* a combination of diethylcarbamazine and albendazole

A recent study carried out in Sri Lanka has confirmed that the chances of detecting hotspots with LF ongoing transmission are higher when the size of the EU is smaller [5]. Our results suggested that the EU size can be reduced by selecting smaller areas with mf- or CFA-positive signals. As such, we would like to propose the following convenient epidemiological approach to find hotspots with ongoing transmission especially in the elimination stage in Sri Lanka (Fig. 4). Mf- or CFA-positive cases will be considered as the index cases. Households and the neighborhood of such index cases can be selected as the MX collection sites. Since our results suggested that the April, May, September, and October are the best months that yield higher *W*. *bancrofti* DNA, MX should be carried out at least in these months. An EU with more than 1% of MX indicates a possible hotspot; therefore, the confirmation can be achieved by demonstrating more than 5% of urine anti-filarial antibody rate among 6 to 7 years schoolchildren of the same EU. Urine anti-filarial antibody testing can be done with ease by selecting schools where the same EU has been identified as the school-catchment area. Once the hotspots are confirmed, it can be removed by MDA using DEC and albendazole (DA) or Ivermectin, DEC, and albendazole (IDA). This approach may not be fast and cost effective like point-of-care tests recommended by WHO. However, this approach remained non-invasive; therefore, sites where blood-based tests are difficult for operation, the said approach can be considered. WHO is in the process of recognizing the value of antibody-based point-of-care tests in the surveillance programme [37]. With WHO recognition, a urine-based antibody test will be prepared as a point-of-care test. Currently, a cheaper urine-based ELISA kit is not commercially available.

## Conclusion

Monthly collections to study LF transmission by MX were conducted for the first time in Sri Lanka. We observed that the filarial DNA-positive rate had an association with seasonal cycle of precipitation. More than 1% filarial DNA and > 5% anti-filarial antibody rates confirmed ongoing transmission in Matotagama. The combination of two non-invasive surveys, the urine anti-filarial IgG4 levels of schoolchildren and MX of vector mosquitoes, would be a convenient package to monitor the ongoing transmission (hotspots) of LF in the surveillance.

## Materials and methods

### Study area and its current transmission status

The town of Matara lies 154 km south of Colombo within the former endemic area of LF. It is the capital of Matara district, where a total of 761,236 people were resident in 2001 [38], and this number increased to 809,236 by 2012 [39]. The present study included several suburbs of Matara town—Walgama (population in 2001; 1760), Walgama North (1820), Walgama Central (1337), and Matotagama (1561) lying within 2 km from the coastline and cover the area of 1.5 km^2^ (Fig. 1). These were highly endemic suburbs during 2001 [40]. All abovementioned suburbs received annual MDA with albendazole and diethylcarbamazine between 2002 and 2006 by the Filariasis Research Training and Service Unit (FRTSU), University of Ruhuna with 100% geographical coverage. A significant reduction of mf counts was observed in all suburbs following 6–12 rounds of MDA and the final round was conducted in 2006 [41]. However, transmission assessment surveys (TAS) conducted in 2008, 2009, 2012, and 2014 had documented residual mf in Matotagama [26]. Matotagama was divided in to two administrative villages due to higher population density as Hamugewatta and Matotagama-subdivision[sd] [41].

### Collection of mosquitoes for filarial DNA detection

Mosquito surveys were carried out in eight houses of Hamugewatta and Matotagama[sd] from February 2013 to June 2014 (Fig. 1). Households with mf-positive cases detected in TAS conducted in 2008 covering both Hamugewatta and Matotagama[sd] were selected as index houses to locate the traps. All mf-positive cases were treated with DEC multi-dose as recommended by the national campaign [41]. A survey conducted in the Hamugewatta and Matotagama[sd] for a different purpose had recorded mf and antigen positives in four of eight indexed houses. All antigen- and mf-positive cases were treated a week after test results [3]. Mosquitoes were captured in gravid traps (CDC Gravid Trap—model 1712) using a liquid bait. The liquid bait was made according to Irish et al. [42]. The gravid traps were placed at the index houses in the evening (5–6 p.m.) and were retrieved in the morning (6–7 a.m.). Precaution was taken to protect the trap from ants and rain. Monthly collections for 17 months were carried out placing the traps at same place in the same households. All trapped mosquitoes were anesthetized using ether before examination under a dissecting microscope to separate *Culex quinquefasciatus* using the key described by Reuben et al. [43]. Next, air-dried female *Cx*. *quinquefasciatus* (fed/unfed and gravid) were pooled into 1.5 mL Eppendorf tubes (1–20 mosquitoes/pool). Each trap collection was pooled separately; therefore, several pools less than 20 mosquitoes were available for analysis.

### Detection of filarial DNA in mosquitoes

DNA was purified from whole bodies of mosquitoes (1–20 in a batch) using QIAamp DNA Mini Kit (QIAGEN, Germany) according to Takagi et al. [44]. Finally, DNA was eluted from the column with 150 μL of Buffer AE. Two microliters of the DNA sample were subjected to PCR. The PCR reaction was performed according to the original report [45, 46]. In brief, the primers NV-1: 5′-CGTGATGGCATCAAAGTAGCG-3′ and NV-2: 5′-CCCTCACTTACCATAAGACAAC-3′ amplified a fragment of 188 bps, a highly repeated DNA sequence in *W*. *bancrofti* genome (SspI repeat). The reaction mixtures in a total volume of 50 μL contained 10 mM Tris–HCl (pH 8.3), 50 mM KCl, 1.5 mM MgCl2, 0.001% (W/V) gelatin, 200 μM of each deoxynucleoside triphosphate, 0.5 μM of each primer, and 1.25 U of AmpliTaq Gold (Applied Biosystems, CA). PCR amplification was performed using a thermal cycler (Takara®, Japan) programmed for 40 cycles of denaturation at 94 °C for 1 min, annealing at 55 °C for 1 min, and extension at 72 °C for 1 min, preceded by an initial denaturation for 5 min at 94 °C. After completion of all cycles, the final extension reaction was for 10 min at 72 °C. The products were analyzed by electrophoresis on a 2% agarose gel in TAE buffer (40 mM Tris acetate, 1 mM EDTA). The gels were stained with ethidium bromide (5 μg/mL) and photographed under ultraviolet illumination.

### Estimation of actual infection rate of mosquitoes

As whole mosquitoes were used in collections to detect the presence of filarial DNA (group testing-20 mosq. in a batch), the number of infected mosquitoes contained in a positive pool should be estimated. The minimum infection rate (MIR) which assumes that a positive group contains only a single infected mosquito apparently underestimates the actual infection rate. Therefore, actual infection rate was estimated based on the total number of pools screened, the number of positive pools observed, and the pool sizes using binGroup library of R software developed by Bilder et al. [29].

### School survey

A list of schools was obtained from the Divisional Educational Office where Hamugewatta and Matotagama[sd] suburbs were documented as the catchment area. Altogether, ten schools were in the list. The top five elementary schools according to the percentage of students from the catchment area (arbitrarily abbreviated—LPV; PMV; WWV; MKV; DSV) in the list were selected for anti-filarial antibody survey. The schoolchildren were in grades 1 to 2, and generally 6 to 7 years old (mean 6.5 years). All schoolchildren of the both grades present on the day were enrolled. The survey was conducted in March 2015. Parents/guardians were requested to collect urine from the schoolchildren in to a wide mouthed plastic cup and transfer 5 mL into a screw-capped plastic sample tube. The samples were mixed with sodium azide at a concentration of 0.1% and stored at 4 °C until their anti-filarial IgG4 titers were measured in immunology laboratory of FRTSU at University of Ruhuna.

### Anti-filarial antibody testing—urine ELISA

Urine ELISA was performed according to previous reports [12, 18, 19]. In brief, 96-well microtiter plates (Maxisorp™; Nunc, Roskilde, Denmark) were coated with recombinant Wb-SXP1 antigen (1 μg/mL) at 4 °C overnight. After blocking with the casein buffer (1% casein in 0.05 M Tris–HCl buffer with 0.15 M NaCl, pH 7.6) for 2 h at room temperature, the plates were directly applied with urine samples (100 μL/well) and incubated overnight at 25 °C. After four washes with phosphate-buffered saline (PBS), pH 7.4 containing 0.05% Tween 20, 100 μL peroxidase conjugated mouse monoclonal antibody to human IgG4 (Southern Biotech, Birmingham, AL), diluted 1:4000, was added to each well. After incubation at 37 °C for 1 h, the plates were washed four times, and then incubated with ABTS® Peroxidase Substrate System (KPL Inc., Gaithersburg, MD) for 1 h at room temperature and the optical density was measured at 415 nm and 492 nm as a reference by Sunrise Rainbow microplate reader (TECAN, Japan). Each sample was assayed induplicate. Antibody levels were expressed as units (U) estimated from a standard curve constructed with serially diluted positive sera in each plate ranging from 0 to 7290 U. The cutoff value in the study was 7.08 U.

### Statistical analysis of the data

We used the Microsoft Excel 2016, and free software R 3.4.4 for the statistical analysis in this paper. The Wilcoxon rank sum test was used to assess the significance of differences in antibody titer (IgG4) distribution among the schools using R software. Filarial DNA-positive rate in mosquitoes (95% confidence intervals for single proportion) were calculated with binGroup library of R software. Partial autocorrelations (ACF) and cross-correlation (CCF) were analyzed to verify the time dependency of several time series variables (monthly rainfall, mosquito population, filarial DNA rate) and existence of cross correlation. ACF is a coefficient that evaluates the time dependency, the correlation with the time difference (lag) for the variable itself. Partial ACF is a coefficient excluding the indirect relationship due to lag 1 stacking. The CCF is a coefficient indicating the similarity between the time series data, and the degree of delay of the cycle is a lag. Box-Ljung test was used for the significance assessment of partial ACF among the collected data. Phillips-Perron test (unit loot test) was performed to show the time series to be stationary. Graphs were produced using ggplot2 and ggfortify library of R software.

### GIS mapping

GPS coordinates were collected for mapping the location of the surveyed five schools and the location of eight mosquito traps. All these location data were plotted on the imported road map (OpenStreetMap) using QGIS 3.2.0 XYZ Tiles function.

### Precipitation data of study area

The precipitation data of Matara was kindly provided by Professor P.L.A.G. Alwis, Department of Agricultural Engineering, University of Ruhuna, Sri Lanka.

## Abbreviations

ACF: Autocorrelation function
CCF: Cross-correlations function
*Cx. quinquefasciatus*: *Culex quinquefasciatus*
GPELF: Global Programme to Eliminate Lymphatic Filariasis
ICT: Immunochromatography test
LF: Lymphatic filariasis
MDA: Mass drug administration
mf: Microfilaria
MX: Molecular xenomonitoring
PCT: Preventive chemotherapy
TAS: Transmission assessment surveys
